# Cancer epidemiology in Central and South Eastern European countries

**DOI:** 10.3325/cmj.2011.52.478

**Published:** 2011-08

**Authors:** Eduard Vrdoljak, Marek Z Wojtukiewicz, Tadeusz Pienkowski, Gyorgy Bodoky, Peter Berzinec, Jindrich Finek, Vladimir Todorović, Nenad Borojević, Adina Croitoru, for South Eastern European Research Oncology Group (SEEROG)

**Affiliations:** 1 Center of Oncology, University Hospital Split, Split, Croatia; 2 Department of Oncology, Medical University of Bialystok, Bialystok, Poland; 3 Clinic of Breast Cancer and Reconstructive Surgery of the Center for Oncology – Maria Curie Memorial Cancer Institute in Warsaw, Warsaw, Poland; 4 St.László Teaching Hospital, Department of Clinical Oncology, Budapest, Hungary; 5 National Oncology Institute, Bratislava, Slovakia; 6 University Hospital, Pilsen, Czech Republic; 7 Oncology Clinic, Clinical Center of Montenegro, Podgorica, Montenegro; 8 Institute for Oncology and Radiology Serbia, Belgrade, Serbia; 9 Clinical Institute Fundeni, Oncology Department, Bucharest, Romania

## Abstract

**Aim:**

To collect cancer epidemiology data in South Eastern European countries as a basis for potential comparison of their performance in cancer care.

**Methods:**

The South Eastern European Research Oncology Group (SEEROG) collected and analyzed epidemiological data on incidence and mortality that reflect cancer management in 8 countries – Croatia, Czech Republic, Hungary, Romania, Poland, Slovakia, and Serbia and Montenegro in the last 20-40 years.

**Results:**

The most common cancer type in men in all countries was lung cancer, followed by colorectal and prostate cancer, with the exception of the Czech Republic, where prostate cancer and colorectal cancer were more common. The most frequent cancer in women was breast cancer followed by colorectal cancer, with the exceptions of Romania and Central Serbia where cervical cancer was the second most common. Cancer mortality data from the last 20-40 years revealed two different patterns in men. In Romania and in Serbia and Montenegro, there was a trend toward an increase, while in the other countries mortality was declining, after increasing for a number of years. In women, a steady decline was observed over many years in the Czech Republic, Hungary, and Slovakia, while in the other countries it remained unchanged.

**Conclusions:**

There are striking variations in the risk of different cancers by geographic area. Most of the international variation is due to exposure to known or suspected risk factors which provides a clear challenge to prevention. There are some differences in incidence and mortality that cannot be explained by exposure to known risk factors or treatment availabilities.

On a global scale, cancer has become a major public health problem and an increasingly important contributor to the burden of disease. Based on the most recent available international data, there were an estimated 12.7 million new cancer cases, 7.6 million deaths from cancer, and 28 million persons alive with cancer within five years from the initial diagnosis (1-3). The most common cancers in the world were lung (1.61 million cases), breast (1.38 million), and colorectal cancer (1.24 million) (3). Because of its poor prognosis, lung cancer was also the most common cause of death (1.38 million), followed by gastric (737 000 deaths), and liver cancer (695 000 deaths) (1-4).

Priority setting for cancer control and cancer services in any region needs to be based on knowledge of the cancer burden and the local mix of predominant cancer types (5). According to estimates of global cancer burden made by the International Agency for Research on Cancer (IARC), the incidence and mortality rates from many specific types of cancer and all cancers combined vary widely by geographic locality (6). Moreover, the IARC also estimated that over half of newly diagnosed cases and two-thirds of cancer deaths occur in low and medium-income countries (6). There are striking variations in the pattern of cancer by site from region to region (7). The large differences in incidence and mortality in different countries may reflect a combination of differences in prevalence of underlying risk factors, differences in host susceptibility, and/or variations in cancer detection, reporting, classification systems, treatment, and follow-up. Among European countries, wide differences in the quality of cancer care are observed, especially when comparison is made between “old” and “new” EU members or between developed and developing countries (8). Cancer survival is significantly lower in Eastern European countries, including the new Member States, than in the EU 15 (9-12). Transitional countries and middle income countries are frequently left forgotten “in between” and the cancer problem in these countries is among the worst and fastest growing (8).

In this report, we provide an analysis, which we propose as a foundation for detailed evaluation of cancer care in selected Central, Southern and Eastern European countries, represented by members of the South Eastern European Research Oncology Group (SEEROG). Our epidemiological analysis indicates the scale of the problem of oncological care in individual countries and shows current trends in the incidence of particular cancers. Comparison of status of oncology between countries in Eastern, Southern, and Central European region has never been undertaken before and key barriers to deliver appropriate quality of care have not previously been identified.

## Material and methods

The methods used to compute the estimates for major cancers are described in detail in GLOBOCAN (1). The basic data used are the best available information on incidence and mortality in selected SEEROG countries. We estimated the incidence and mortality rates for selected Southern and Eastern European countries by sex and cancer site, for four age groups (0-19, 20-44, 45-64, 65+ years) using the most recent data. The countries included in the analysis were Croatia, Czech Republic, Hungary, Poland, Romania, Slovakia, and Serbia and Montenegro. Estimates of the resident population of individual countries for the years 2002 to 2006, generally based on official censuses, were obtained from the World Health Organization (WHO) database. Age-standardized incidence and mortality rates per 100 000 populations were calculated. From the matrices of certified deaths and resident population numbers, incidence and mortality rates were calculated for specific age groups (0-19, 20-44, 45-64, 65+ years) and time periods. The world standard population was used for calculations (13).

Historical data on cancer mortality rates covered the period from the early 1960s to the first decade of the 21st century. The time frame for the individual countries was: Croatia 1985-2006; Czech Republic 1970-2005; Hungary 1965-2005; Poland 1965-2006; Romania 1969-2004; Serbia and Montenegro 1997-2002 (although Serbia and Montenegro are now two independent countries, we present results for both countries together as over this period they were one state); and Slovakia 1971-2005. The data for specific tumor sites were abstracted from WHO database, including the number of deaths by sex and 5-year age groups according to cancer site in the relevant years (14). Mortality data were collected for all cancers and for selected cancer sites by searching for the following International Classification of Diseases version 10 (ICD-10) codes: cancer general (C00-C97), stomach (C16), colorectal (C18-C21), lung (C33-C34), breast (C50), uterine cervix (C53), ovary (C56), prostate (C61), kidney (C64), and urinary bladder (C67). For long-term observations, the source data were re-coded into ICD-10 according to the recommendations of the ICD and WHO committee (15).

Sources of data on incidence are much more disparate. Attempts were made to collect data for the whole of each country. The sources of data and time frames were as follows:

1. Croatia: Croatian National Cancer Registry, Croatian National Institute of Public Health, Zagreb; 1993-2006 (16);

2. The Czech Republic: Czech National Cancer Registry; 1977-2005 (17);

3. Hungary: Globocan 2002 (process of creating national cancer register has been started but it ended with project phase) (1);

4. Poland: National Cancer Register; 1980-2006 (18);

5. Romania: Globocan 2002 (there are some local registers; however, these registers significantly underestimated incidence; until 2007, registration was at the hospital and clinic level and there were no national institution collecting data) (1).

6. Serbia: Cancer Registry of Central Serbia, Institute of Public Health of the Republic of Serbia (75% of Serbian population) 2002-2004 (19).

7. Montenegro: there is no reliable source of data on cancer incidence in Montenegro as a whole, but the data were derived from cancer registries covering a part of the country (19).

8. Slovakia: National Cancer Registry, Cancer Research Institute SAS, Bratislava; 1978-2003 (20).

## Results

The analysis covered a total population of 98 million people in all the participating countries. The population sizes of the chosen countries ranged from 4.5 million in Croatia to 38 million in Poland (Table 1).

**Table 1:** Population characteristics of the selected countries: Croatia, Czech Republic, Hungary, Poland, Romania, Serbia and Montenegro, and Slovakia

Country	Year of data collection	Population (in thousands)		
		male	female	total
**Croatia**	**2005**	2138.6	2303.3	4441.9
**Czech Republic**	**2005**	4991.4	5242.7	10 234.1
**Hungary**	**2005**	4788.8	5298.2	10 087.1
**Poland**	**2006**	18 436.1	19 696.2	38 132.3
**Romania**	**2004**	10 571.6	11 101.7	21 673.3
**Serbia and Montenegro**	**2002**	3947.1	4161.5	8108.7
**Slovakia**	**2005**	2615.9	2773.3	5389.2

### Incidence

There were almost 360 000 newly diagnosed cancer patients per year in the study region. The greatest number based on the size of populations was found in Poland (n=126 019) and Croatia (n=20 186) (Table 2).

**Table 2:** Age-standardized incidence rates for all cancers by sex and country – Croatia, Czech Republic, Hungary, Poland, Romania, Central Serbia, and Slovakia (last available year)

Country	Year of data collection	Male						Female					
		n	0-19	20-44	45-64	65+	all ages	n	0-19	20-44	45-64	65+	all ages
**Croatia**	2005	11 030	19.1	71.1	655.4	2255.2	314.2	9156	16.3	94.2	506.4	1078.8	210.3
**Czech Republic**	2005	28 147	12.7	64.4	761.5	2676.7	359.0	26 627	12.0	170.4	647.0	1439.1	286.4
**Hungary**	2002	26 278	11.5	78.7	916.1	2504.9	386.8	22 924	9.1	93.8	582.9	1381.7	250.6
**Poland**	2006	64 092	13.8	46.0	553.3	1818.5	253.6	61 927	12.1	76.6	506.5	923.1	191.8
**Romania**	2002	32 244	12.3	53.9	544.9	1226.1	216.4	27 655	9.9	84.0	397.3	696.2	163.4
**Central Serbia**	2004	12 801	21.7	84.3	692.8	1674.7	286.2	11 954	17.1	134.4	640.4	1036.5	246.7
**Slovakia**	2003	12 159	16.9	73.2	807.4	2662.5	371.4	11 841	12.5	95.9	627.5	1443.4	257.9

Detailed analysis by age showed that in children (0-19 years) the highest cancer incidence was observed in Central Serbia (21.7/100 000 for boys and 17.1/100 000 for girls) and Croatia (19.1/100 000 and 16.3/100 000, respectively). The lowest incidences were recorded in Hungary (11.5/100 000 and 9.1/100 000, respectively) and Romania (12.3/100 000 and 9.9/100 000, respectively). In this age group, cancer was diagnosed more frequently in boys than in girls, by approximately 20% (Table 2).

Cancer incidence in young adults (20-44 years) was significantly higher in women than in men in most countries. Incidence rates in women were between 76.6/100 000 in Poland and 170.4/100 000 in Czech Republic, which is a much greater difference than between the rates in men in that age group. The incidence in men was highest in Central Serbia (84.3/100 000) and lowest in Poland (46.0/100 000) (Table 2).

In the middle-aged population (45-64 years), cancer incidence was about 30% higher in men than in women. The highest incidence in men was found in Hungary (916.1/100 000) and the lowest in Poland (553.3/100 000) and Romania (544.9/100 000). The highest cancer incidence in women in this age group was in the Czech Republic, Central Serbia, and Slovakia (≥627.5/100 000). The lowest incidence was estimated for Romania (397.3/100 000) (Table 2).

In the elderly population (≥65 years), cancer was diagnosed almost twice as often in men as in women. The highest incidence in older men was found in the Czech Republic (2676.7/100 000) and Slovakia (2662.5/100 000) and the lowest in Romania (1226.1/100 000). The pattern of cancer incidence in older women was the same as in older men: the highest in Slovakia (1443.4/100 000) and the Czech Republic (1439.1/100 000), and the lowest in Poland (923.1/100 000) and Romania (696.2/100 000) (Table 2).

Table 3 shows age-standardized incidence rates for the selected most common cancer sites.

**Table 3:** Age-standardized incidence rates for selected cancers (available year) by country Croatia, Czech Republic, Hungary, Poland, Romania, Serbia and Montenegro, and Slovakia

Country	Year of data collection	Male						Female							
		lung	stomach	large bowel	prostate	kidney	urinary bladder	lung	stomach	large bowel	breast	cervix	ovary	kidney	urinary bladder
**Croatia**	**2005**	63.7	17.7	43.5	38.7	11.1	18.4	13.6	6.9	23.4	55.8	9.2	11.7	4.7	4.1
**Czech Republic**	**2005**	58.2	11.2	58.7	59.5	22.6	22.3	15.5	5.8	28.8	61.7	13.3	12.4	9.8	6.0
**Hungary**	**2002**	94.6	20.5	56.6	34.0	14.7	19.6	24.9	9.5	33.7	63.0	15.7	11.1	6.6	5.1
**Poland**	**2006**	60.2	12.8	29.3	27.3	9.1	15.9	14.5	4.9	17.2	44.5	11.5	11.1	4.7	3.0
**Romania**	**2002**	50.0	17.6	22.0	16.8	5.5	15.4	8.5	6.8	14.4	44.3	23.9	9.4	2.8	3.3
**Central Serbia**	**2004**	64.2	12.9	33.7	20.1	6.5	17.4	18.4	7.2	20.9	57.9	24.3	10.9	3.8	4.2
**Slovakia**	**2004**	55.8	16.1	55.5	33.9	14.9	15.9	10.4	7.0	27.7	49.7	15.2	11.5	6.6	4.1

**Figure 1:**
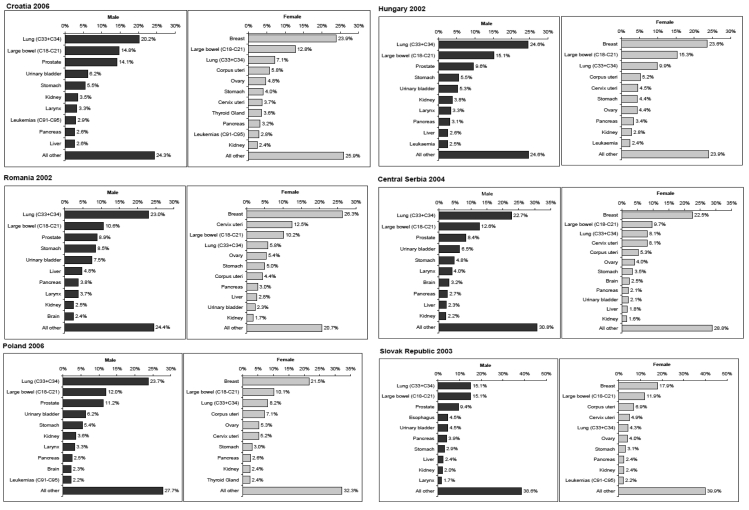
Overview of cancer incidence by site and sex for selected countries – Croatia, Hungary, Romania, Central Serbia, Poland, and Slovakia.

Lung cancer was the most common cancer in men in all studied countries, with the exception of the Czech Republic, where prostate cancer and colorectal cancer were more common. The second most frequent cause of cancer morbidity was colorectal cancer followed by prostate cancer, which was ranked third in most countries, with the highest proportion in the Czech Republic and the lowest proportion in Central Serbia. The incidence of kidney cancer ranged from ~ 2% in most countries to 6.3% in the Czech Republic. The latter is a special case, with a kidney cancer incidence 2-4 times higher than other countries in the region. The most frequent cancer in women was breast cancer, with the highest incidence in Romania and the lowest in Slovakia. The second most common tumor was colorectal cancer. An exception was Romania, where cervical cancer was the second most common cancer in women. In the third place, there was lung cancer (Croatia, Hungary, Poland, and Central Serbia), uterine cancer (Czech Republic, Slovakia), or colorectal cancer (Romania). Kidney cancer was responsible for 2-4% of the cancer incidence (highest in the Czech Republic). Figure 1 gives an overview of cancer incidence by site, sex, and country.

### Mortality

The greatest number of cancer deaths was observed in Poland (91 632), which had the biggest population of the studied countries and the fewest cancer deaths occurred in Slovakia (n=11 794) and Croatia (n=12 639), attributed to the smaller size of the populations (Table 4).

**Table 4:** Cancer mortality compared to total mortality by country – Croatia, Czech Republic, Hungary, Poland, Romania, Serbia and Montenegro, and Slovakia

Country	Year of data collection	Population (in thousands)			Number of total deaths			All cancer deaths			Frequency of cancer deaths (%)		
		male	female	total	male	female	total	male	female	total	male	female	total
**Croatia**	**2005**	2138.6	2303.3	4441.9	26 058	25 720	51 778	7430	5209	12 639	29	20	24
**Czech Republic**	**2005**	4991.4	5242.7	10 234.1	54 072	53 866	107 938	15 567	12 466	28 033	29	23	26
**Hungary**	**2005**	4788.8	5298.2	10 087.1	69 773	65 950	135 723	17 134	13 481	30 615	25	20	23
**Poland**	**2006**	18 436.1	19 696.2	38 132.3	198 298	171 388	369 686	51 777	39 855	91 632	26	23	25
**Romania**	**2004**	10 571.6	11 101.7	21 673.3	138 440	120 450	258 890	25 641	18 037	43 678	19	15	17
**Serbia and Montenegro**	**2002**	3947.1	4161.5	8108.7	111 108	105 350	216 458	11 216	8330	19 546	10	8	9
**Slovakia**	**2005**	2615.9	2773.3	5 389.2	28 151	25 324	53 475	6906	4888	11 794	25	19	22

The proportion of deaths attributable to cancer was highest for men in Croatia and the Czech Republic (29%), while in Poland and Hungary about a quarter of deaths were caused by cancer. The highest proportion of cancer deaths in women was found in Poland and the Czech Republic (23%), while in other countries (except Romania, and Serbia and Montenegro) cancer deaths constituted a fifth of all deaths (Table 4).

Table 5 presents cancer mortality data by age group. In the 0-19 years age group, the highest cancer mortality in boys was found in Romania (6.5/100 000) and Serbia and Montenegro (5.6/100 000) and in girls in Romania (4.8/100 000). The lowest cancer mortality in this age group was recorded in the Czech Republic and Hungary (2.2-3.4/100 000). In the 20-44 years age group, the highest cancer mortality in men was observed in Hungary (30.8/100 000) and Romania (28.1/100 000). Women had the highest cancer mortality in Romania (30.9/100 000), Serbia and Montenegro (29.2/100000), and Hungary (28.9/100000). In the other countries, the cancer mortality rates were similar and about 50% lower. In this age group, cancer mortality rates in women were equal to or higher than rates in men. Significantly higher cancer mortality rates in men aged 45-64 years were observed in Hungary, with the rate of 563.8/100 000 being 30%-50% higher than in other countries. Similarly in the female population, mortality was highest in Hungary (275.0/100 000) and lowest in Romania (210.7/100 000) and Croatia (197.6/100 000). Cancer mortality in elderly men (≥65 years) was similar in Croatia, the Czech Republic, Poland, and Slovakia (1550-1675/100 000). Cancer mortality was about 30%-35% lower ( ~ 1100/100 000) in Romania and Serbia and Montenegro. In women, a similar pattern was observed, with the lowest mortality in Serbia and Montenegro and Romania.

**Table 5:** Standardized mortality rates from all cancers in selected countries – Croatia, Czech Republic, Hungary, Poland, Romania, Serbia and Montenegro, and Slovakia by age group and sex

Country	Male					Female				
	0-19	20-44	45-64	65+	all ages	0-19	20-44	45-64	65+	all ages
**Croatia**	3.9	22.5	410.6	1674.7	204.4	3.8	19.6	197.6	746.4	98.0
**Czech Republic**	2.6	17.7	375.1	1643.5	193.4	2.2	19.3	223.3	856.4	109.8
**Hungary**	3.4	30.8	563.8	1530.3	226.1	2.7	28.9	275.0	785.7	118.1
**Poland**	4.3	19.3	396.1	1602.9	195.7	3.5	20.2	243.8	725.9	105.4
**Romania**	6.5	28.1	404.6	1082.7	164.8	4.8	30.9	210.7	560.1	91.6
**Serbia and Montenegro**	5.6	25.9	388.3	1148.3	165.2	4.0	29.2	249.9	624.0	102.7
**Slovakia**	4.1	20.7	428.1	1594.1	201.6	4.0	18.9	218.3	707.9	99.1

In women, the highest cancer mortality was found in Hungary (118.1/100 000) and the Czech Republic (109.8/100 000) and the lowest in Romania (91.6/100 000) and Croatia (98/100 000) (Table 5).

Table 6 presents cancer mortality data by selected, the most common cancer sites. In most countries, the highest proportion of cancer mortality in men was attributed to lung cancer, followed by colorectal cancer and prostate cancer. In Romania, the ranking was different, with stomach cancer in the second place. Similarly, in Serbia and Montenegro, stomach cancer was the third cause of cancer mortality, followed by prostate cancer. The next positions among the ten most frequent cancers are occupied by pancreatic cancer, bladder cancer, liver cancer, laryngeal cancer, leukemia, esophageal cancer, and kidney cancer. Esophageal cancer was in the first ten in the Czech Republic, Slovakia, and Hungary, while kidney cancer was in the first ten in Croatia, Czech Republic, Poland, and Slovakia. In women, breast cancer had a dominant position in most countries. The proportion of cancer deaths due to breast cancer was between 13.1% (Poland) and 17.7% (Croatia). However, in two countries other cancer types caused more deaths in women: lung cancer and colorectal cancer in Hungary and colorectal cancer in Slovakia. Colorectal cancer and lung cancer were in second place, causing a similar proportion in cancer mortality. The next positions were taken by pancreatic cancer, ovarian cancer, and stomach cancer. In Romania, cervical cancer was the third most common cause of cancer deaths. This phenomenon was not observed in other countries.

**Table 6:** Age-standardized selected cancers mortality rates in selected countries (Croatia, Czech Republic, Hungary, Poland, Romania, Slovakia in 2005, and Serbia and Montenegro in 2002)

Country	Male						Female							
	lung	stomach	large bowel	prostate	kidney	urinary bladder	lung	stomach	large bowel	breast	cervix	ovary	kidney	urinary bladder
**Croatia**	55.3	14.3	25.0	15.3	4.9	6.3	10.7	5.9	12.0	17.4	2.5	5.7	1.8	1.2
**Czech Republic**	52.4	9.3	30.0	16.3	9.2	5.9	12.8	4.6	14.2	17.7	3.9	7.4	3.5	1.5
**Hungary**	72.5	12.4	31.9	13.0	5.4	6.9	22.3	5.8	16.4	19.2	4.9	5.6	2.2	1.6
**Poland**	64.6	13.6	18.8	12.9	5.8	8.0	14.3	4.9	10.8	14.9	5.7	7.0	2.2	1.3
**Romania**	48.2	15.4	15.6	9.0	2.8	5.7	8.5	5.3	9.1	16.6	10.7	5.4	1.1	1.1
**Serbia and Montenegro**	51.9	11.5	18.4	9.5	2.9	6.2	12.3	4.7	10.1	19.3	6.9	4.4	1.6	1.1
**Slovakia**	50.5	12.0	30.6	14.9	5.9	5.4	7.8	5.7	13.3	15.3	5.1	5.9	2.7	1.2

**Figure 2:**
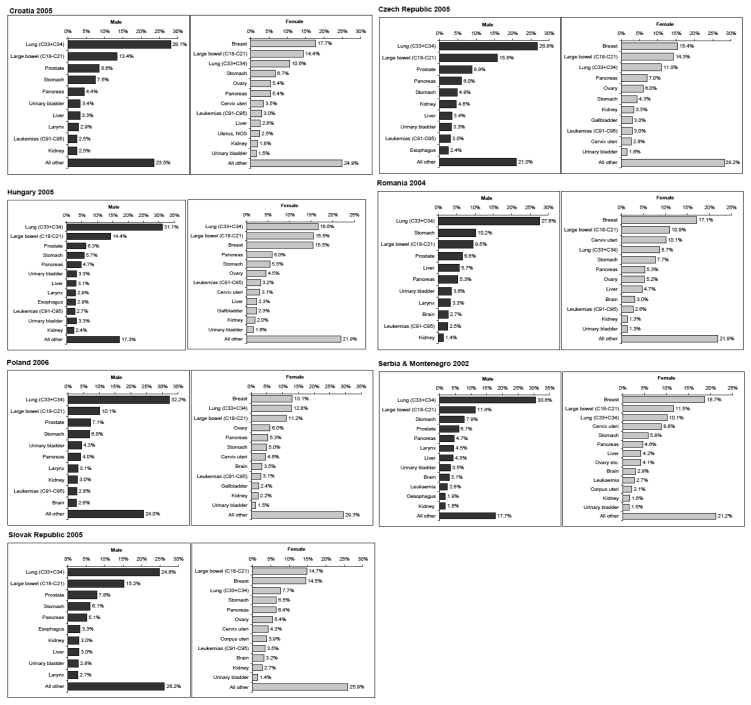
Cancer deaths for 10 cancer sites by sex for selected countries – Croatia, Czech Republic, Hungary, Romania, Central Serbia, Poland, and Slovakia.

The distribution of cancer deaths in men and women by site of tumor is presented in Figure 2.

**Figure 3:**
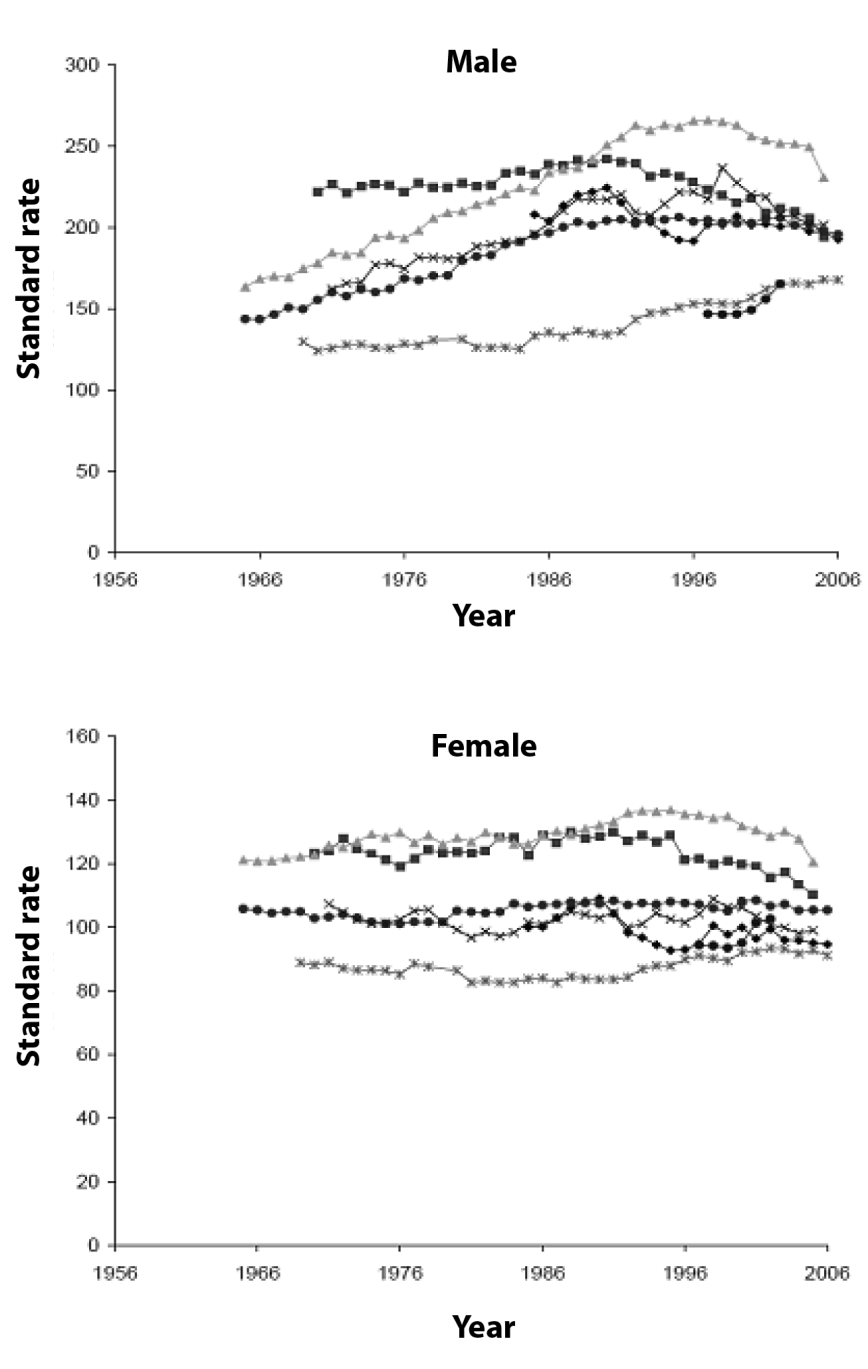
Mortality from all cancers over time in selected countries by sex; black triangle – Hungary, black square – Czech Republic, black circle – Poland, black cross – Slovakia, black rhomb – Croatia, white circle – Serbia and Montenegro, gray cross – Romania.

Mortality trends from all cancers in the last 20-40 years (depending on data available) are shown in Figure 3.

Cancer mortality trends in the last 20-40 years show that there are two conflicting patterns in men. In Romania and Serbia and Montenegro, an increasing trend was observed while in other countries, after many years of increasing cancer mortality, rates were decreasing. The most significant decrease in cancer mortality was recorded in the Czech Republic and Slovakia. In the female population, an improving trend was observed in the Czech Republic, Hungary, and Slovakia, with a constant decrease in cancer mortality over many years in these countries. In other countries, cancer mortality in women remained unchanged.

## Discussion

There are striking variations in cancer epidemiology and in quality of cancer care in different regions of the world (7). Differences in quality of cancer care have been observed between European countries, especially when “old” and “new” EU member states are compared (11,21,22). This study provides updated estimates of cancer epidemiology in eight selected Central, South and East European countries. Several sources of information and different methods have been used to generate these statistics. In general, the cancer problem is worst and fastest growing among developing countries and hence prompt action is needed using all known anticancer strategies, from primary prevention to early detection and adoption of the best available anticancer treatments (21). As mentioned, this article describes the cancer epidemiology situations in selected Central, South and Eastern European countries in order to address the specific problems and needs of different countries.

The highest cancer incidence in men was found in Hungary (386.8/100,000), while the rates in Poland, Central Serbia, and Croatia were lower by a third and in Romania by half (Table 2). In women, the highest incidence rates were found in the Czech Republic (286.4/100 000) and Slovakia (257.9/100 000). The lowest incidence, below 200/100 000, was noted in Poland and Romania. Unfortunately, the observed age-standardized incidence rates are among the highest in the world, underlining the urgent need to invest more in the fight against cancer (1,3).

Detailed analysis by age showing differences in the incidence of cancer in the age group 0-19 years of almost 50% in such a small region and between relatively similarly developed countries implies possible differences in the prevalence of underlying risk factors and differences in host susceptibility. However variations in cancer detection, reporting, classification, treatment, and follow-up may be even more important. For example, Romania has one of lowest incidence rates but the highest mortality rates, implying either inadequate reporting of cancer cases (lack of cancer register in Romania) or challenges in cancer treatment in patients in this age group.

The observation that cancer incidence in young adults (20-44 years) is significantly higher in women than in men in most countries is in line with similar observation from Western Europe (1). But, in some countries such as the Czech Republic differences are three times higher, which warrants further investigations. Nevertheless, despite huge differences in incidence between the sexes, the mortality rates are similar, indicating the fact that women have more treatable tumors and better survival rates. In the middle-aged population (45-64 years), cancer incidence was about 30% higher in men than in women. Such difference in incidence rates results in even greater difference in mortality, meaning that men have double chance of dying of cancer than women. In the elderly population (≥65 years), cancer was diagnosed almost twice as often in men as in women. Cancer mortality in elderly men (≥65 years) was similar in Croatia, the Czech Republic, Poland, and Slovakia (1550-1675/100 000). It was about 20% lower ( ~ 1100/100 000) in Romania and Serbia and Montenegro. In women, a similar pattern was observed, with the lowest mortality in Serbia and Montenegro and Romania.

In the former 15 EU countries, mortality trends for all cancer sites were more favorable than in Central and Eastern European accession countries that entered the EU in 2004 and 2007, including Hungary, Czech Republic, Poland, Slovakia, and Romania (10,11). In particular, these countries had the highest rates not only for lung cancer and other tobacco-related cancers, but also for gastric and cervical cancers and leukemias (10-12). Moreover, for all cancer sites, trends were more favorable in the former 15 EU countries than in these countries. According to Boyle et al, maintenance and potential improvement of favorable trends in cancer mortality would require a strategy focusing on the control of tobacco and alcohol consumption, nutrition and diet, and avoiding excessive sun exposure (23). Unfortunately, the highest tobacco consumption in Europe is in its Eastern part and highest alcohol consumption in the central part, particularly in the Czech Republic in which the average daily consumption was 56.9 g/L in men and 14.6g/L in women (24). For selected cancer sites, early diagnosis can also have a relevant impact, and this together with the universal adoption of recent therapeutic advances in countries from Central, South, and Eastern Europe may contribute to reducing the cancer mortality burden, as was most probably the case with cervical cancer mortality in Romania and Central Serbia (10,11). In 2000, total cancer mortality for men in the EU was 165.6/100 000, whereas total cancer mortality rates in the eight Central and Eastern European accession countries ranged from 194.5/100 000 in Lithuania to 269.3/100 000 in Hungary (10). The median cancer mortality rate in men for all countries in this SEEROG analysis was 193.02/100 000 (from 164.8/100 000 in Romania to 226.1/100 000 in Hungary). This cancer mortality rate was significantly higher than in five Western European countries (median 146.9/100 000 in 2005); the rates by country are: 152.4/100 000 in Spain, 163.6/100 000 in France, 138.7/100 000 in the UK, 136.4/100 000 in Ireland, and 143.2/100 000 in Germany (10). The cancer mortality rates for women in the SEEROG countries ranged from 91.6/100 000 in Romania to 118.1/100 000 in Hungary, while in the five Western European countries the rates ranged from 69.4/100 000 in Spain to 105.8/100 000 in Ireland. The median cancer mortality rate in the SEEROG countries was 103.52/100 000, compared with 89.72/100 000 in five Western European countries.

The most important factors that influence cancer mortality rates are adequate cancer screening, diagnosis, and treatment (10,25). Also, and no less important, is the distribution of different types of cancer. Namely, while prostatic cancer is becoming the most common type of cancer in developed countries, lung cancer, a significantly more deadly tumor type, is the most prevalent cancer in the developing world. The excess mortality from these neoplasms in Eastern European countries could therefore be reduced if adequate resources, training, and logistics to deliver adequate diagnosis and treatment were implemented (26-29).

## References

[1] Ferlay J, Bray F, Pisani P, Parkin DM. GLOBOCAN 2002: cancer incidence, mortality and prevalence worldwide. IARC CancerBase No. 5. version 2.0. Lyon (France): IARC Press; 2004.

[2] Ferlay J, Autier P, Boniol M (2007). Cancer Incidence and mortality in Europe, 2006.. Ann Oncol.

[3] Ferlay J, Shin HR, Bray F, Forman D, Mathers C, Parkin DM. GLOBOCAN 2008 v1.2: Cancer incidence and mortality worldwide: IARC CancerBase No. 10. Lyon (France): IARC Press; 2010. Available from: http://globocan.iarc.fr Accessed: July 29, 2011.

[4] American Cancer Society. Cancer facts and figures 2008, American Cancer Society, Atlanta. Available from: http://www.cancer.org/downloads/STT/2008CAFFfinalsecured.pdf Accessed: June 05, 2009.

[5] Parkin DM, Bray F, Ferlay J, Pisani P (2001). Estimating the world cancer burden: Globocan 2000.. Int J Cancer.

[6] Berrino F, Sant M, Verdecchia A, editors. Survival of cancer patients in Europe: The EUROCARE Study. Lyon (France): IARC Scientific Publication; 1995.

[7] Parkin DM, Bray F, Ferlay J, Pisani P (2005). Global cancer statistics, 2002.. CA Cancer J Clin..

[8] MEPs against cancer. Cancer facts and figures. Available from: http://www.mepsagainstcancer.org Accessed: July 26, 2011.

[9] Levi F, Lucchini F, Negri E, Boyle P, La Vecchia C (2004). Cancer mortality in Europe, 1995–1999, and an overview of trends since 1960.. Int J Cancer.

[10] Levi F, Lucchini F, Negri E, Zatonski W, Boyle P, La Vecchia C (2004). Trends in cancer mortality in the European Union and accession countries, 1980-2000.. Ann Oncol.

[11] Levi F, Lucchini F, Negri E, La Vecchia C (2004). Trends in mortality from major cancers in the European Union, including acceding countries.. Cancer.

[12] Ferlay J, Autier P, Boniol M, Heanue M, Colombet M, Boyle P (2007). Estimates of the cancer incidence and mortality in Europe in 2006.. Ann Oncol.

[13] Doll R, Smith PG. Comparison between registries: age-standardized rates. Vol. IV. In: Waterhouse JAH, Muir CS, Shanmugaratnam K, editors. Cancer incidence in five continents. Lyon (France): IARC Scientific Publications; 1982. p. 671-5.

[14] World Health Organization Statistical information System. WHO mortality database. Available from: http://www3.who.int/whosis/whosis/menu.cfm Accessed: July 26, 2011.

[15] World Health Organization international statistical classification of diseases and related health problems, 10th revision. Geneva (Switzerland): World Health Organization; 1992.

[16] Croatian National Cancer Registry, Croatian National Institute of Public Health, Zagreb. Annual reports 1993-2006. Available from: http://www.hzjz.hr/cancer/index.htm*.* Accessed: July 26, 2011.

[17] Czech National Oncologic Register (NOR), Institute of Health Information and Statistics. (UZIS CR). 1977-2005. Available from: http://www.svod.cz/?sec=analyzy Accessed: July 26, 2011.

[18] Poland – National Cancer Register. 1980-2006; Available from: http://epid.coi.waw.pl/krn*.* Accessed: July 26, 2011.

[19] Cancer Registry of Central Serbia, Institute of Public Health of the Republic of Serbia 2002-2004. Available from: http://www.batut.org.rs/*.* Accessed: July 26, 2011.

[20] Slovakia – National Cancer Registry, Cancer Research Institute SAS, Bratislava. 1978-2003. Available from: http://www.exon.sav.sk/web/index.php*.* Accessed: July 26, 2011.

[21] Vrdoljak E, Wojtukiewicz MZ, Pienkowski T, Bodoky G, Berzinec P, Finek J (2008). Current oncology situation in Eastern and Southern European countries: South Eastern European Research Oncology Group (SEEROG) Initiative, ESMO 2008. Annals of Oncology.

[22] Marmot M, Bobak M (2000). International comparators and poverty and health in Europe.. BMJ.

[23] Boyle P, Autier P, Bartelink H, Baselga J, Boffetta P, Burn J (2003). European code against cancer and scientific justification: third version.. Ann Oncol.

[24] Boniol M, Autier P (2010). Prevalence of main cancer lifestyle risk factors in Europe in 2000.. Eur J Cancer.

[25] Levi F, Lucchini F, Franceschi S, Negri E, La Vecchia C (2001). Inequalities in health in Europe.. BMJ.

[26] Levi F, Lucchini F, Boyle P, Negri E, La Vecchia C (2003). Testicular cancer mortality in Eastern Europe.. Int J Cancer.

[27] Boyle P (2004). Testicular cancer: the challenge for cancer control.. Lancet Oncol.

[28] Janssens JP, Giacosa A, Stockbrugger R (2003). The European community expansion and cancer burden.. Eur J Cancer Prev.

[29] Antunes JLF, Toporcov TN, De Andrade FP (2003). Trends and patterns of cancer mortality in European countries.. Eur J Cancer Prev.

